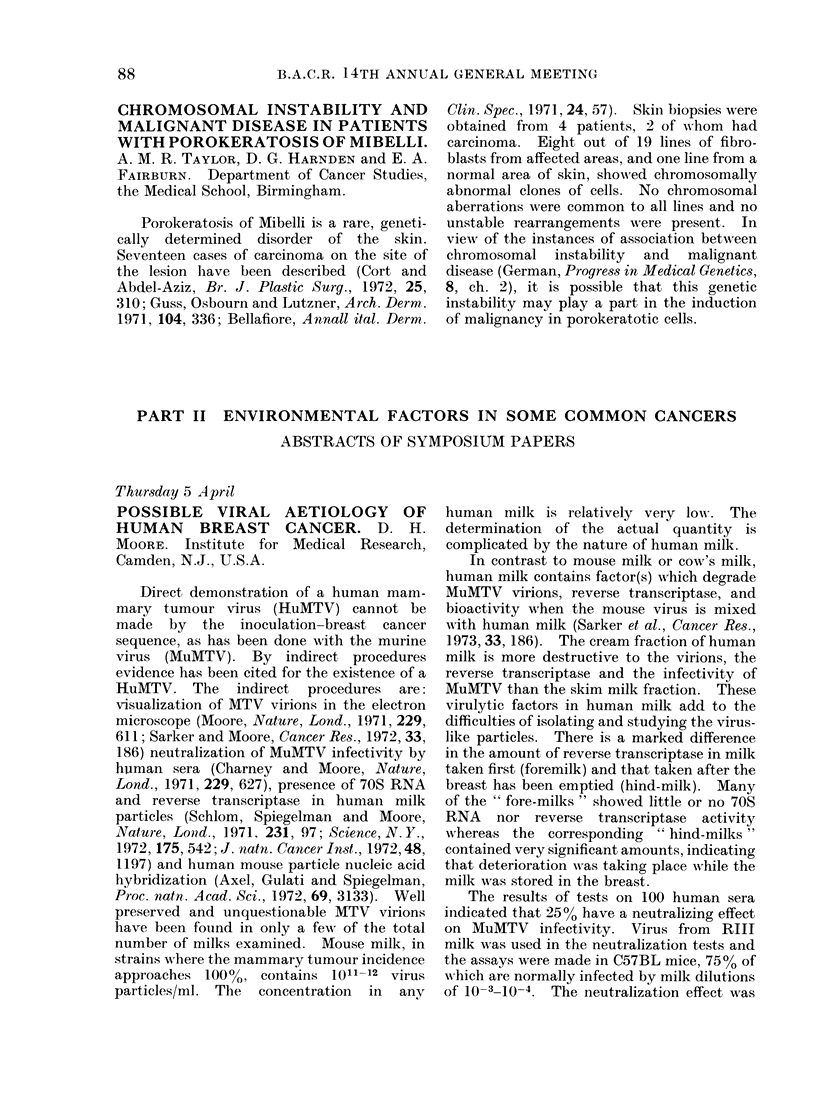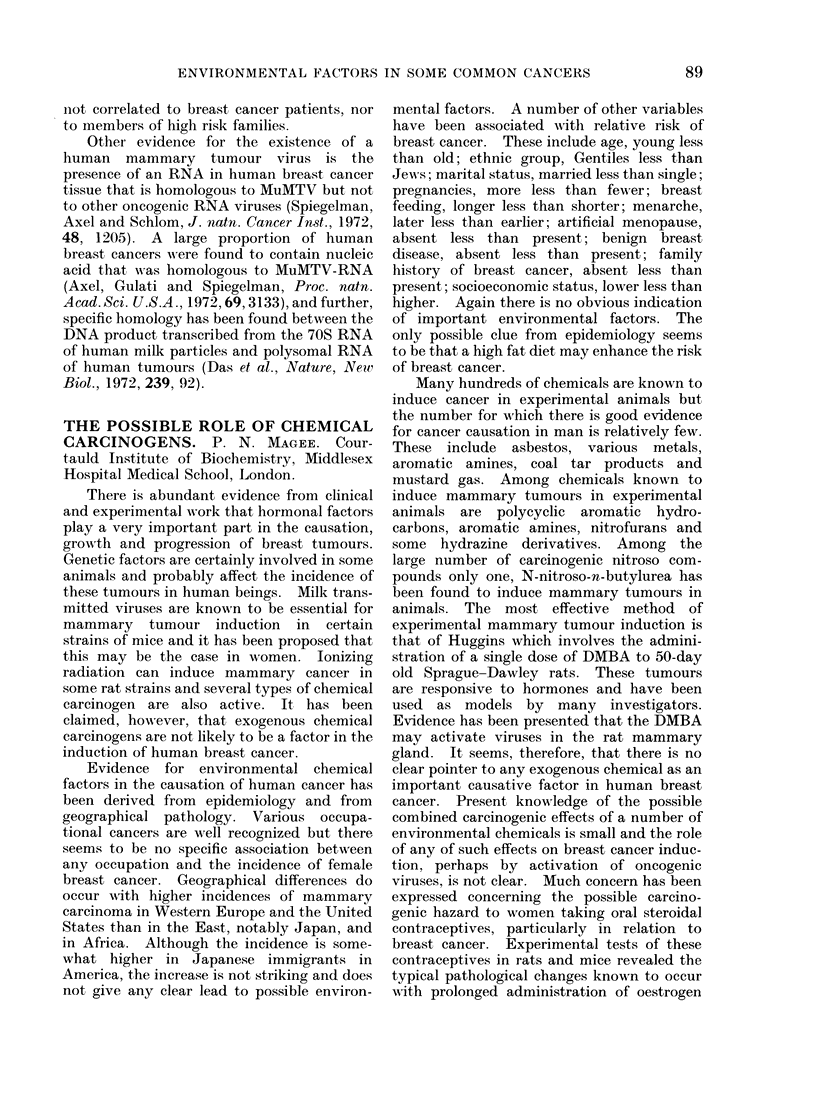# Possible viral aetiology of human breast cancer.

**DOI:** 10.1038/bjc.1973.115

**Published:** 1973-07

**Authors:** D. H. Moore


### PART II ENVIRONMENTAL FACTORS IN SOME COMMON CANCERS

### ABSTRACTS OF SYMPOSIUM PAPERS

*Thursday 5 April*

**POSSIBLE VIRAL AETIOLOGY OF HUMAN BREAST CANCER.** D. H. MOORE. Institute for Medical Research, Camden, N.J., U.S.A.

Direct demonstration of a human mammary tumour virus (HuMTV) cannot be made by the inoculation–breast cancer sequence, as has been done with the murine virus (MuMTV). By indirect procedures evidence has been cited for the existence of a HuMTV. The indirect procedures are: visualization of MTV virions in the electron microscope (Moore, *Nature, Lond.*, 1971, **229**, 611; Sarker and Moore, *Cancer Res.*, 1972, **33**, 186) neutralization of MuMTV infectivity by human sera (Charney and Moore, *Nature, Lond.*, 1971, **229**, 627), presence of 70S RNA and reverse transcriptase in human milk particles (Schlom, Spiegelman and Moore, *Nature, Lond.*, 1971, **231**, 97; *Science, N.Y.*, 1972, **175**, 542; *J. natn. Cancer Inst.*, 1972, **48**, 1197) and human mouse particle nucleic acid hybridization (Axel, Gulati and Spiegelman, *Proc. natn. Acad. Sci.*, 1972, **69**, 3133). Well preserved and unquestionable MTV virions have been found in only a few of the total number of milks examined. Mouse milk, in strains where the mammary tumour incidence approaches 100%, contains $10^{11-12}$ virus particles/ml. The concentration in any human milk is relatively very low. The determination of the actual quantity is complicated by the nature of human milk.

In contrast to mouse milk or cow's milk, human milk contains factor(s) which degrade MuMTV virions, reverse transcriptase, and bioactivity when the mouse virus is mixed with human milk (Sarker *et al.*, *Cancer Res.*, 1973, **33**, 186). The cream fraction of human milk is more destructive to the virions, the reverse transcriptase and the infectivity of MuMTV than the skim milk fraction. These virulytic factors in human milk add to the difficulties of isolating and studying the virus-like particles. There is a marked difference in the amount of reverse transcriptase in milk taken first (foremilk) and that taken after the breast has been emptied (hind-milk). Many of the " fore-milks " showed little or no 70S RNA nor reverse transcriptase activity whereas the corresponding " hind-milks " contained very significant amounts, indicating that deterioration was taking place while the milk was stored in the breast.

The results of tests on 100 human sera indicated that 25% have a neutralizing effect on MuMTV infectivity. Virus from RIII milk was used in the neutralization tests and the assays were made in C57BL mice, 75% of which are normally infected by milk dilutions of $10^{-3}$–$10^{-4}$. The neutralization effect was not correlated to breast cancer patients, nor to members of high risk families.

Other evidence for the existence of a human mammary tumour virus is the presence of an RNA in human breast cancer tissue that is homologous to MuMTV but not to other oncogenic RNA viruses (Spiegelman, Axel and Schlom, *J. natn. Cancer Inst.*, 1972, **48**, 1205). A large proportion of human breast cancers were found to contain nucleic acid that was homologous to MuMTV-RNA (Axel, Gulati and Spiegelman, *Proc. natn. Acad. Sci. U.S.A.*, 1972, **69**, 3133), and further, specific homology has been found between the DNA product transcribed from the 70S RNA of human milk particles and polysomal RNA of human tumours (Das *et al.*, *Nature, New Biol.*, 1972, **239**, 92).